# A Sub-Acute Dosing Study of Saxitoxin and Tetrodotoxin Mixtures in Mice Suggests That the Current Paralytic Shellfish Toxin Regulatory Limit Is Fit for Purpose

**DOI:** 10.3390/toxins15070437

**Published:** 2023-07-03

**Authors:** Sarah C. Finch, Nicola G. Webb, Michael J. Boundy, D. Tim Harwood, John S. Munday, Jan M. Sprosen, Chanatda Somchit, Ric B. Broadhurst

**Affiliations:** 1 AgResearch Ltd., Ruakura Research Centre, Private Bag 3123, Hamilton 3240, New Zealand; nikki.webb@agresearch.co.nz (N.G.W.); jan.sprosen@agresearch.co.nz (J.M.S.); chanatda.somchit@agresearch.co.nz (C.S.); ric.broadhurst@agresearch.co.nz (R.B.B.); 2 Cawthron Institute, Private Bag 2, Nelson 7042, New Zealand; michael.boundy@cawthron.org.nz (M.J.B.); tim.harwood@cawthron.org.nz (D.T.H.); 3 Department of Pathobiology, School of Veterinary Science, Massey University, Private Bag 11 222, Palmerston North 4442, New Zealand; j.munday@massey.ac.nz

**Keywords:** toxicology, paralytic shellfish toxins, feeding study, saxitoxin, tetrodotoxin, dosing protocols

## Abstract

Paralytic shellfish poisoning is a worldwide problem induced by shellfish contaminated with paralytic shellfish toxins. To protect human health, a regulatory limit for these toxins in shellfish flesh has been adopted by many countries. In a recent study, mice were dosed with saxitoxin and tetrodotoxin mixtures daily for 28 days showing toxicity at low concentrations, which appeared to be at odds with other work. To further investigate this reported toxicity, we dosed groups of mice with saxitoxin and tetrodotoxin mixtures daily for 21 days. In contrast to the previous study, no effects on mouse bodyweight, food consumption, heart rate, blood pressure, grip strength, blood chemistry or hematology were observed. Furthermore, no histological findings were associated with dosing in this trial. The dose rates in this study were 2.6, 3.8 and 4.9 times greater, respectively, than the highest dose of the previous study. As rapid mortality in three out of five mice was observed in the previous study, the deaths are likely to be due to the methodology used rather than the shellfish toxins. To convert animal data to that used in a human risk assessment, a 100-fold safety factor is required. After applying this safety factor, the dose rates used in the current study were 3.5, 5.0 and 6.5 times greater, respectively, than the acute reference dose for each toxin type set by the European Union. Furthermore, it has previously been proposed that tetrodotoxin be included in the paralytic shellfish poisoning suite of toxins. If this were done, the highest dose rate used in this study would be 13 times the acute reference dose. This study suggests that the previous 28-day trial was flawed and that the current paralytic shellfish toxin regulatory limit is fit for purpose. An additional study, feeding mice a diet laced with the test compounds at higher concentrations than those of the current experiment, would be required to comment on whether the current paralytic shellfish toxin regulatory limit should be modified.

## 1. Introduction

Paralytic shellfish poisoning (PSP) is caused by the ingestion of paralytic shellfish toxins (PSTs) which can accumulate in filter-feeding shellfish. These toxins are generated by the marine dinoflagellates *Alexandruim*, *Gymnodinium* and *Pyrodinium* [1,2,3]. The toxin class responsible for PSP is composed of saxitoxin (STX) and over 50 structural analogues [4]. The symptoms of PSP start with numbness and tingling of the lips and mouth which may progress to muscle weakness, incoordination, and neurological symptoms [5]. Symptoms such as muscular paralysis will be present in advanced cases and death may occur due to respiratory paralysis [6,7]. PSP is a significant worldwide issue that has been reported throughout history. The first documented case was in 1793 when the captain of a ship recorded a poisoning event in his diary [8], and in Alaska between 1973 and 1994 there were 54 outbreaks, 117 PSP cases, 29 emergency treatments and 1 death [9].

To protect human health, shellfish must be tested for PSTs to determine whether they are safe for human consumption. Traditionally, this was done using a mouse bioassay (MBA) in which a shellfish extract was injected into a mouse, and if it died within a set timeframe, the shellfish was deemed to be unsafe [10]. Due to the large numbers of mice required to adequately monitor shellfish using this method, it was considered to be unethical and has now been replaced by analytical methods [11]. However, the MBA yields the overall toxicity of a sample irrespective of the STX analogues present, whereas, although different analogues can be detected and quantified analytically, this does not correlate with toxicity, since the analogues have a range of toxicities. This can be handled using toxicity equivalence factors (TEFs) which compare the toxicity of STX to that of each analogue [12]. The TEFs determined in this way can then be applied to the concentrations of each analogue; evaluated analytically, allowing the total concentration to be calculated; and expressed as STX equivalents (STX eq). Although the toxicity of each STX analogue must be determined using mice, the numbers required for this testing are a fraction of those previously used in the MBA. Tetrodotoxin (TTX) is a seafood toxin that shares the same mode of action as the PSP toxins and which induces human intoxication with the same symptoms [13]. Although the presence of TTX in pufferfish and other related fish species has long been acknowledged [14], it is becoming more prevalent in shellfish species [15,16]. Currently, TTX is not regulated, and the European Union (EU) manages the risk by banning the sale of fish species known to be associated with TTX accumulation [17]. However, international regulators now recognize TTX as an emerging threat. Due to their common mechanism of action, it has been proposed that TTX be included in the suite of PSP toxins [13]. A recent study that showed the toxicity of TTX and STX.2HCl to be additive, both by intraperitoneal injection (i.p) and by oral administration, demonstrated that this proposal is valid [18].

To assess the safety of a shellfish sample, the analytically determined PST concentration must be compared to that which has been deemed safe for human consumption, the regulatory limit. A regulatory limit has been adopted by many countries, with the current limit set by the EU at 800 µg STX.2HCl eq/kg shellfish flesh (regulation (EC) No 853/2004) [6], and the same limit was recommended at the twenty-eighth session of the Codex Committee on Fish and Fishery Products (CCFFP) in 2006 [19]. This resulted in the development of the Standard for Live and Raw Bivalve Molluscs (CODEXSTAN 292-2008, rev 2015) [20]. These limits are expressed in terms of STX dihydrochloride (STX.2HCl) because this is more stable than the free base (STX). Confusion can arise in the literature if it is not stated whether concentrations are expressed in terms of STX or STX.2HCl, and adoption of standard units has been proposed [21]. The regulatory limit is obviously vital to keep consumers safe, but in addition, this level also impacts the shellfish industry, since their product must adhere to this standard. It is therefore critical that the regulatory limit is based on robust and high-quality data.

The most appropriate data would be that generated from human poisoning events. However, to determine the dose rate responsible for illness, three different pieces of information are required: (1) The PST concentration of the food consumed. It is critical that remnant food, rather than shellfish retrospectively gathered from the same area is analysed, since PST concentrations can change quickly [22]. In addition, PST concentrations may be affected by the cooking process and whether the broth is included in the meal, has a significant impact on toxicity [23,24]. (2) The meal size consumed. (3) The bodyweight of the consumer. It is very rare that all of these three necessary pieces of data are documented, meaning that animal data plays a key role in the determination of toxicity and the setting of regulatory limits. However, on the basis of available human data, the European Food Safety Authority (EFSA) determined that the acute reference dose (ARfD), defined as the quantity of STX which could be consumed within a 24 h period without causing adverse effect, was 0.5 µg STX.2HCl eq/kg bodyweight [6]. EFSA defines a large portion size as 400 g of shellfish flesh [25] and uses a figure of 60 kg for the bodyweight of an adult [6]. If a 60 kg human ate 400 g of shellfish contaminated with PSTs at the current regulatory limit (800 µg STX.2HCl eq/kg shellfish flesh), they would be exposed to a dose rate of 5.3 µg/kg STX.2HCl [6]. However, a more recent EFSA document has suggested that 70 kg may be a more realistic adult bodyweight [26]. The use of this figure would reduce the human dose rate to 4.6 µg/kg STX.2HCl.

Another consideration is that shellfish may be consumed by humans on a regular basis, and as such, experiments using repeated dosing of mice with toxins is required. A previous study fed mice either a control diet or a diet containing one of three concentrations of STX.2HCl for 21 days [27]. To mimic human feeding behaviour, mice were fed meals by allowing unrestricted access to food for two 1 h periods per day. This trial showed no adverse effects from STX.2HCl up to a dose rate of 715 µg/kg/day [27]. To extrapolate animal data to that which can be used in a human risk assessment, safety factors must be used. To account for the species difference, a 10-fold safety factor is applied, and to account for any possible variation of susceptibility within a human population, a further 10-fold safety factor is applied [28]. Applying these safety factors to the dose rate which was determined to induce no adverse effects in mice yields a safe dose rate of 7.15 µg/kg/day, which is greater than the 5.3 µg/kg generated by EFSA using the current PSP regulatory limit (800 µg STX.2HCl eq/kg shellfish flesh), equivalent to a human with a low bodyweight (60 kg) consuming a large portion size (400 g) [6]. Since the data used by EFSA represents a worst-case scenario, Finch et al. [27] were able to conclude that the current PSP regulatory limit was appropriate.

However, in contrast to the Finch et al. [27] study, Boente-Juncal et al. [29] fed low-concentration mixtures of STX.2HCl/TTX to mice for 28 days and observed a high level of toxicity. There were two major differences between the conflicting trials. First, Boente-Juncal et al. [29] dosed their subjects with a mixture of TTX and STX.2HCl, whereas Finch et al. [27] dosed theirs with STX.2HCl alone. Although it has been shown that the acute toxicities of TTX and STX.2HCl are additive [18], it is possible that this may not be the case with repeated dosing with the two toxins. The other difference was in the method of administration. In the Boente-Juncal et al. [29] study, a daily one-off bolus dose of the toxins was administered by gavage, whereas the study by Finch et al. [27] incorporated the toxin into the diet of the mice, which was made available for two 1 h periods per day. This method was chosen to mimic human feeding behaviour.

Due to the importance of the PSP regulatory limit in protecting human health and facilitating international trade it is vital that this limit is accurate. The objective of this study was therefore to investigate the apparent differences between the two trials detailed above. In this study we report the results of a dosing study using an oral, once-a-day bolus dose of TTX and STX.2HCl mixtures at three different concentrations. Mice were dosed daily for 21 days, and food intake, bodyweight, motor control, blood pressure, heart rate, grip strength, blood chemistry and haematology were measured. In addition, histology was performed on tissues and organs. The results of this study could then be used to establish whether the PSP regulatory limit appeared fit for purpose.

## 2. Results

### 2.1. Appearance and Behaviour

No changes in behaviour were observed throughout the trial. Despite appearing healthy, with normal growth and food intakes in the days prior, 4 deaths were observed in the high treatment group (days 2, 6, 13 and 17) (325/162.5 µg/kg STX.2HCl/TTX) and one death was noted in the medium dose treatment group (day 9) (250/125 µg/kg STX.2HCl/TTX). These mice became hunched and lethargic 1–2.5 h post-dosing with their ears back. The lethargy worsened over time, and the hind legs of mice became splayed. Death was observed 3–6 h post-dosing, which was characterized by a slowing of respiration and jerky, running movements of the back legs. Since there were no indications of any effect of the toxins prior to death and all the remaining mice in the treatment groups were indistinguishable from mice in the control group, the deaths were attributed to a dosing artefact caused by the toxins being absorbed through the buccal membranes.

### 2.2. Bodyweight

**Figure 1:**
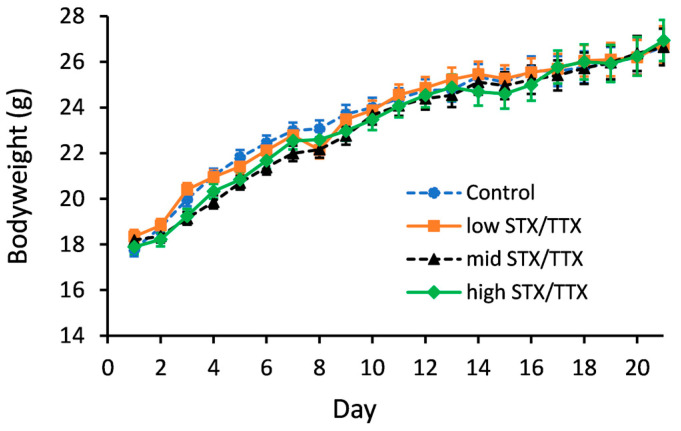
Temporal trend in bodyweights of mice dosed with control (- -●- -), 175/87.5 µg/kg STX.2HCl/TTX (low) (--■--), 250/125 µg/kg STX.2HCl/TTX (medium) (- -▲- -) or 325/162.5 µg/kg STX.2HCl/TTX (high) (--♦--) daily for 21 days. The error bars denote the standard error of the predicted bodyweights.

Statistical analysis of the bodyweight data showed that there was no evidence of any interaction between gender and treatment (gender.treatment.day, *p* = 0.171; gender.treatment, *p* = 0.587). A graph could therefore be created for the temporal treatment effect pooled over gender (Figure 1).

Statistical analysis showed that on days 4, 6 and 7, the mean bodyweight of mice in the medium STX/TTX treatment group was less than that of the control group (*p* < 0.05). On day 5, mice dosed with the medium STX/TTX mixture and the high STX/TTX mixture had a lower bodyweight than the mice in the control group (*p* < 0.05). There were no other statistically significant differences between the mean bodyweights of mice dosed with the control matrix and those dosed with any of the three dose rates of STX/TTX throughout the 21-day experimental period.

### 2.3. Food Intake

**Figure 2:**
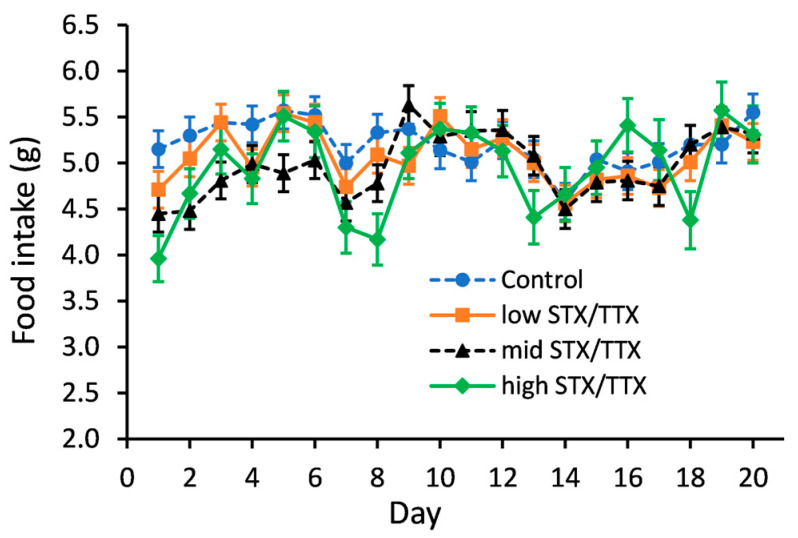
Temporal trend in food intake of mice fed control (- -●- -), 175/87.5 µg/kg STX.2HCl/TTX (low) (--■--), 250/125 µg/kg STX.2HCl/TTX (medium) (- -▲- -) or 325/162.5 µg/kg STX.2HCl/TTX (high) (--♦--) daily for 21 days. The error bars denote the standard error of the predicted food intake.

Statistical analysis of food intake data showed that there was no evidence of an interaction between gender and treatment (gender.treatment.day, *p* = 0.360; gender.treatment, *p* = 0.343). A graph could therefore be created for the temporal treatment effect pooled over gender (Figure 2).

Statistical analysis showed that on days 1, 2, 3 and 5, the mean food intake of mice in the medium STX/TTX treatment group was lower than that of the control group (*p* < 0.05). On days 1 and 8, mice dosed with the high STX/TTX dose rate had a lower food intake compared to mice of the control group (*p* < 0.05). There were no other statistically significant differences between the mean food intake of mice dosed with the control matrix and those dosed with any of the three dose rates STX/TTX throughout the 21-day feeding period.

### 2.4. Motor Coordination

**Figure 3:**
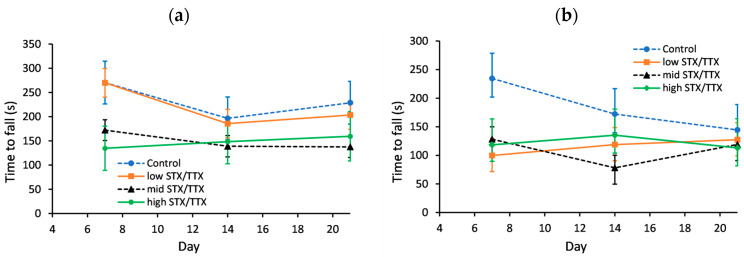
Predicted time to fall for (**a**) female mice and (**b**) male mice dosed with control matrix (- -●- -), 175/87.5 µg/kg STX.2HCl/TTX (low) (--■--), 250/125 µg/kg STX.2HCl/TTX (medium) (- -▲- -) or 325/162.5 µg/kg STX.2HCl/TTX (high) (--♦--) daily for 21 days. The error bars denote the standard error of the predicted time to fall.

The motor coordination of each mouse was measured on days 0, 7, 14 and 21 using an accelerating rotarod. The day 0 data was used as a covariate in the statistical analysis. This analysis showed a gender.treatment.day interaction (*p* < 0.05), so the genders were analysed separately (Figure 3).

For female mice, the only statistically significant difference in motor coordination was on day 7, when mice dosed with the control matrix took longer to fall than mice fed STX/TTX at the medium and high dose rates.

On day 7, the time to fall of male mice was greater for the control group in comparison to any of the other three treatments (*p* < 0.05), while on day 14 the time to fall was lower for mice dosed with STX/TTX at the medium dose rate in comparison to the control.

### 2.5. Grip Strength

**Figure 4:**
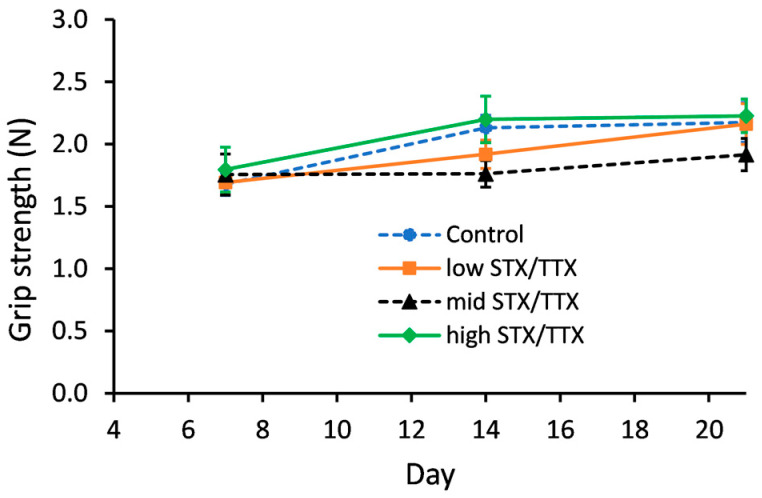
Temporal trend in grip strength of mice fed control matrix (- -●- -), 175/87.5 µg/kg STX.2HCl/TTX (low) (--■--), 250/125 µg/kg STX.2HCl/TTX (medium) (- -▲- -) or 325/162.5 µg/kg STX.2HCl/TTX (high) (--♦--) daily for 21 days. The error bars denote the standard error of the predicted grip strength.

Grip strength was measured on days 0, 7, 14 and 21 using a grip strength meter. The data collected on day 0 was used as a covariate in the statistical analysis. This analysis showed that there was no evidence of an interaction between gender and treatment (gender.treatment.day, *p* = 0.439; gender.treatment, *p* = 0.410). A graph could therefore be created for the temporal treatment effect pooled over gender (Figure 4).

There were no statistically significant differences between the grip strength of mice dosed with the control matrix and those dosed with STX/TTX at any of the three different dose rates on any of the measurement days.

### 2.6. Heart Rate and Blood Pressure

Heart rate and blood pressure data (Table 1) were collected only on days 14 and 21 since, prior to this time, the mice were too small to fit into the blood pressure analysis system. Statistical analysis of heart rate and blood pressure showed that there was no evidence of any interaction between gender and treatment, allowing the data to be combined.

There were no statistically significant differences in the heart rates or systolic or diastolic blood pressures of mice dosed with the control matrix and those dosed with STX.2HCl/TTX at any of the three dose rates (*p* > 0.05).

### 2.7. Haematology

Results of the haematological analysis of blood collected on day 21 are presented in Table 2. There was no evidence of an effect of gender on any of the analytes measured (*p* > 0.05), allowing the data to be pooled.

There were no statistically significant differences between any of the analytes measured in the blood samples taken from mice of the different treatment groups.

### 2.8. Blood Chemistry

Results of the serum biochemical analysis of blood collected from mice on day 21 is presented in Table 3. There was no evidence of any effect of gender on any of the parameters measured (*p* > 0.05), allowing the genders to be pooled.

For all the parameters measured, there were no statistically significant differences between the mice dosed with the control matrix and those dosed with high STX/TTX. The only statistically significant difference between the control and any of the STX/TTX treatment groups was for ALT in the medium STX/TTX group.

### 2.9. Organ Weights

The organ weights of all mice, expressed as percentage of bodyweight, are presented in Table 4.

The kidney weights of the female mice fed the high STX/TTX diet were the lowest of the treatment groups, whereas the male mice in the high STX/TTX group had high kidney weights. The log kidney weights of female mice were statistically significantly higher for mice in the control group in comparison to any of the mice fed STX/TTX. However, no difference in log kidney weights was observed between the male control and any of the STX/TTX treatments. Consideration of the other statistically significant differences observed for the organ weights, expressed as a percentage of bodyweight, shows the male mice in the high STX/TTX treatment groups had higher brain weights compared to those of control mice. However, no statistical difference in log brain weights was observed between the female control and any of the STX/TTX-treated females. Although not statistically significant, the log brain weights of female mice fed the high STX/TTX diet had the lowest brain weight of the four treatment groups, which was the opposite of what was observed in male mice. There was no statistical evidence of any effect of treatment, gender, or their interactions on heart and liver weights (*p* > 0.05). For spleen weights, there was no statistical evidence of any effect of interactions between treatment and gender, allowing the data to be pooled. The mice in the low STX/TTX treatment group showed statistically significant differences in log spleen weights compared to the mice in the control group. The multiple comparison for the treatment effect indicated that the mice in the low STX/TTX treatment group showed a statistically significant difference in log spleen weights compared to the mice in the control group. However, the log spleen weights of mice in the medium and high STX/TTX treatment groups were not significantly different from those of mice in the control group. This effect on spleen weight demonstrated a lack of dose dependency and is therefore unlikely to be toxicologically significant.

### 2.10. Histological Examination

For mice in all treatment groups, sections of brain, heart, kidneys, liver, spleen, adrenals, lungs, pancreas, gastrocnemius, jejunum, ovary/uterus or testes, stomach, thymus and urinary bladder were examined and found to be within normal histological limits. Additionally, multiple sections of spinal cord were examined without identification of histological abnormalities.

### 2.11. Summary

To be confident of a true toxicologically significant difference between mice dosed with a test compound and those dosed with a control matrix, the effect on the measured parameter would be expected to be observed in both genders. In addition, it would be expected that the treatment effect shows a dose dependency, hence the use of multiple dose rates in toxicological testing. The statistically significant differences seen in this experiment do not meet this criterion. Furthermore, although 5 mice died during the experiment, apparently due to a dosing artefact, all remaining mice were perfectly healthy. It therefore appears highly unlikely that STX/TTX up to a dose rate of 325/162.5 µg/kg, given as a once-a-day bolus dose for 21 days, induced any adverse effects.

## 3. Discussion

The major objectives of this study were to investigate the contrasting results of two previous sub-acute PST dosing studies and to assess whether the current PSP regulatory limit is appropriate. The two previous studies were performed by Boente-Juncal et al. [29] and Finch et al. [27]. In the former study, toxicity was reported at a dose rate of 44 µg/kg for TTX and 54 µg/kg STX.2HCl, whereas in the latter, dosing with STX.2HCl induced no toxicity at dose rates up to 715 µg/kg. There were two major differences between the two trials; in the Boente-Juncal et al. study [29], a once-a-day gavage dose was administered, whereas Finch et al. [27] incorporated the STX into normal mouse diet. The other difference was that Boente-Juncal et al. [29] used a TTX/STX mixture whereas Finch et al. [27] used only STX. To be able to effectively compare results, the current study utilized STX/TTX mixtures. In addition, although the incorporation of the test compound into the diet better mimics human feeding behaviour, in this case a once-a-day dosing protocol was employed. However, gavage dosing was avoided as it is often associated with accidental administration of the dosing material into the lungs, which can result in rapid death. In addition, both gavage-related reflux and mechanically induced reflux are possibilities [30]. A study by Craig and Elliott [31], utilizing a radiolabeled protein, showed that 38% of mice did not receive the appropriate dose when it was administered by gavage, leading the authors to conclude, “the common method of gavage feeding mice to assess absorption of orally ingested material can lead to artifacts not seen when the same agent is consumed under more natural circumstances”. In acute toxicity studies, the incorporation of the test compound with a small amount of cream cheese (150–200 mg) has worked very successfully [32,33]. However, this is not appropriate for a repeated-dose study, since consumption is voluntary and over time mice may refuse to consume the dose, especially if they start to feel ill. As an alternative, we used a method whereby a very small amount of ground mouse food was combined with the toxin and dosed over the tongue of the mouse, a method which has been used successfully in the past [34]. Since mice cannot spit or vomit, if administered correctly, the dose is swallowed or retained in the mouth. However, prior to the experiment, eight of the ten mice dosed with the high dose rate of STX/TTX died with symptoms consistent with PSP. In contrast, a further ten mice given the same dose rate but with cream cheese applied to their whiskers post-dosing showed no adverse effects. This methodology, which induced immediate grooming, the production of saliva and swallowing was therefore used in the study. Previous studies have shown that cream cheese has no impact on the toxicity of PSTs [35]. However, we did see deaths in our experiment as described in Section 2.1. This was unexpected, as the mice in the high dose treatment group (4 deaths) had a combined STX/TTX dose rate of 487.5 µg/kg/day and the mice in the medium dose treatment group (1 death) had a combined STX/TTX dose rate of 375 µg/kg/day. In contrast, the Finch et al. feeding study [27] showed no toxicity at STX dose rates of up to 715 µg/kg/day, and the acute oral LD_50_ of STX and TTX as a one-off bolus dose is 1060 µg/kg. For all 5 affected mice in the current study, no signs of toxicity were observed in the days prior to death. Of particular importance were the food intake data, since a reduced food intake is a consistent sign of PST toxicity in mice. Furthermore, the surviving mice in each treatment group showed no signs of toxicity and all health parameters measured were normal. We therefore hypothesize that these deaths were because a portion of the dose was retained in the mouth rather than being swallowed. Since PSP toxins are known to be absorbed through the buccal membranes [36], this would result in rapid absorption and an overestimation of toxicity. The dosing performed prior to the experiment was also consistent with this hypothesis. When dosed with STX/TTX at the high dose concentration (325/162.5 µg/kg for STX and TTX, respectively) mice which were induced to swallow (cream cheese mixture was applied to their whiskers) showed no adverse effects, whereas on dosing with the same toxin concentration, a high death rate was observed in mice which were not induced to swallow. The deaths observed in the 21-day dosing study are therefore believed to be attributable to a dosing artefact. Since the current study was conducted, primarily, to investigate the trial of Boente-Juncal et al. [29], a once-a-day dosing regime was necessary. However, a better study would be one wherein the toxins are incorporated into the diet of mice, as this would eliminate any possibility of a dosing artefact.

The dose rates chosen for our study were based on the EFSA ARfDs for STX.2HCl and TTX, which are 0.5 and 0.25 µg/kg, respectively. Adding the 100-fold safety factor to allow animal data to be extrapolated to humans gives dose rates of 50 µg/kg STX.2HCl and 25 µg/kg TTX for animals. The dose rates used in our study were 3.5 (low dose), 5.0 (medium dose) and 6.5 (high dose) times the ARfD for each compound (175, 250 and 325 µg/kg STX.2HCl and 87.5, 125 and 162.5 µg/kg TTX). A comparison of the highest dose rates used in this study with those in the study conducted by Boente-Juncal et al. [29] found the former to be 6.0 (STX.2HCl) and 3.6 (TTX) times higher. The dose rates used by Boente-Juncal et al. [29] appear to have been chosen using some false assumptions. The dose rate of TTX used was 44 µg/kg, which was stated to be that of the ARfD. However, this figure is the regulatory limit of TTX in shellfish flesh rather than the AfRD. The STX.2HCl dose rates used in Boente-Juncal et al. [29] were said to be based on the maximal exposure level of 5.3 µg/kg STX.2HCl (5.3, 17, 54 µg/kg/day for low, medium and high dose treatment groups) derived by EFSA [6]. However, safety factors were not applied to allow the extrapolation of animal data to humans. As a result, the dose rates of STX.2HCl used were considerably lower than the EFSA-derived figure (100, 31 or 10 times lower for low, medium and high dose rates, respectively). Although we used much higher dose rates in our current study, we saw no adverse effects, whereas in the Boente-Juncal et al. study [29], one quarter of the mice in their low dose treatment group (combined STX/TTX dose of 49.3 µg/kg/day) and two fifths of the mice in their high dose treatment group (combined STX/TTX dose of 98 µg/kg/day) died. Given that these dose rates are 4.7 and 9.2% of the acute oral LD_50_ and 2.1 and 4.0% of the acute oral no observable adverse effect level (NOAEL) of STX and TTX, this was surprising. Deaths were documented as “sudden convulsions and rapid death”, and it was stated that “these findings are consistent with the clinical signs of toxicity induced by STX and TTX where death is associated with jerky and running movements of the back legs”. While it is true that the symptomology is characteristic of STX and TTX poisoning, the rapid death time is not. In the study referenced by Boente-Juncal et al. [29], the onset of symptoms by gavage administration of TTX or TTX/STX mixtures is reported to be up to 2 ½ h, while death was observed 1–5 h post-dosing [18]. The very rapid deaths reported in the Boente-Juncal et al. [29] study are more consistent with accidental dosing of the toxin mixture into the lungs of the mouse. Consistent with this hypothesis, the mice showed no adverse effects in the days leading up to their deaths and had normal growth. Food intake was also normal, whereas it has previously been shown that a reduced food intake is a consistent sign of PSP in mice [35]. Furthermore, the survivors showed no symptoms of PSP and remained normal throughout the trial. Issues associated with gavage dosing are well-known, as described previously. The toxicity induced by low dose rates in the Boente-Juncal et al. study [29], along with the very rapid death times of the mice, suggest that the Boente-Juncal et al. study [29] may be flawed.

To assess the current regulatory limit, we can take the highest dose rate used in our toxicological study, which showed no adverse effects, and apply the 100-fold safety factor to allow the extrapolation of animal data to a human risk assessment. This showed that the dose rates of TTX and STX.2HCl were each 6.5 times their respective ARfD. Acute toxicity data has shown that the toxicities of TTX and STX are additive, and because they share the same mode of action, it has been proposed that TTX be added to the suite of PSP toxins [18]. The highest dose rate used in our experiment was 13 times greater than the ARfD. For a 70 kg human to reach the corrected (after safety factors are applied) combined dose rate given to mice, 427 g of shellfish flesh contaminated at the current regulatory limit (800 µg/kg STX.2HCl eq/kg shellfish flesh) would need to be consumed. This quantity of shellfish is greater than a large portion size as defined by EFSA (400 g) [25]. To allow a direct comparison with the Boente-Juncal et al. results [29], the current study used a daily bolus dose of toxin, whereas incorporation of the toxin into the diet of mice and using a mealtime feeding protocol better mimics human feeding behaviour. Dosing with STX.2HCl using the latter experimental protocol showed no adverse effects even at a dose rate 1.5 times that of the current study [27].The results of this study showed that the Boete-Juncal et al. experiment [29] appeared to be flawed and so is not appropriate for use in the discussion of the current PSP regulatory limit. Consistent with the previous study, where STX.2HCl was administered alone, the dosing of a TTX and STX.2HCl mixture to mice in the current study indicates that the current PSP regulatory limit appears fit for purpose. However, a further study wherein mice are fed the toxins as part of their normal diet, using dose rates of STX/TTX higher than those used in the current trial, would be more appropriate for assessing the accuracy of the PSP regulatory limit. Such a study would eliminate the possibility of dosing artefacts and would be more relevant to the consumption of PSTs by humans.

## 4. Materials and Methods

### 4.1. Purity Assessment of Saxitoxin and Tetrodotoxin

STX standard (National Research Council of Canada (NRC)) was used to calibrate the STX used in this study (Cawthron Institute, Nelson, NZ, USA) using HPLC-UV [27]. Trace concentrations of other PSTs were detected and quantified using LC-MS/MS [37]. The STX used in this study was 99.8% pure with 0.16% decarbamoylSTX and 0.05% neoSTX. The STX stock solution (10.91 mg/mL STX.2HCl in 3 mM HCl) was stored at 4 °C.

TTX citrate free (10 mg; Cayman Chemicals, purchased from Sapphire Bioscience) was dissolved in 10 mL of 10 mM acetic acid. This solution was calibrated against certified reference material from the NRC and from Cifga (Spain) as described by Finch et al. [18].

STX/TTX working solutions (in 3 mM HCl) were prepared gravimetrically.

### 4.2. Animals

Swiss albino mice were used for all experimental work and were individually caged in a temperature-controlled room (21 ± 1 °C). Mice had unrestricted access to water and were exposed to a 12-h light–dark cycle. All animal manipulations were approved by the Ruakura Animal Ethics Committee established under the Animal Protection (Code of Ethical Conduct) Regulations Act, 1987 (New Zealand). The project number for this study was 15296, and the approval date was 4 March 2021. During the feeding study, boxes containing individual mice were randomised in columns and rows such that the eight combinations of treatment group and gender occurred exactly once along each row (forming the experimental replicate) and no more than once down each column.

### 4.3. Preparation of STX/TTX for Dosing

Dosing was performed by applying the STX/TTX dose over the tongue of the mouse. Teklad Global 2016 mouse food pellets (Harlan UK, Bicester, England) were ground to a fine flour using a cyclone sample mill (Udy Corporation, Fort Collins, CO, USA). A small amount of this material (approximately 20 mg) was placed in the glass tip of a positive displacement pipette. A 10 µL aliquot of the appropriate STX/TTX dosing solution was then added and mixed using a thin wire to yield a firm paste. In preparation for the experiment, 10 mice were dosed with the high concentration of STX/TTX using this method, which surprisingly resulted in 8 deaths with symptomology consistent with PSP. It was hypothesized that this was because a portion of the dose was retained in the mouth rather than being swallowed, resulting in adsorption through the buccal membranes and an overestimation of toxicity. To induce swallowing, a small blob of 1:1 cream cheese–water (approximately 25 mg) was applied to the whiskers of the mice after the administration of the test dose. Using this method, no deaths were observed on dosing 10 mice with the same high STX/TTX dose rate. This protocol was therefore adopted for the 21-day dosing trial.

### 4.4. Sub-Acute 21-Day Dosing Trial

Two days prior to the experiment, 30 female and 30 male weanling mice were individually caged. On each of these two days, mice were pre-trained on an accelerating rotarod (Rotamex 4/8, Columbus Instruments International, Columbus, OH, USA). Mice were put on the rod which accelerated from 13 to 79 rpm over 12 min. Each mouse was tested twice and the time to fall recorded. Any mouse which did not adapt to the test was excluded from the study. From the remaining mice, 20 females and 20 males were selected and randomly assigned to treatment groups. There were four treatment groups, and in accord with OECD guideline 407 [38], each group contained five female and five male mice (individually caged). Group 1—daily dose of control matrix (3 mM HCl), Group 2—low dose STX/TTX daily (175 and 87.5 µg/kg for STX.2HCl and TTX, respectively), Group 3—medium dose STX/TTX daily (250 and 125 µg/kg for STX.2HCl and TTX, respectively) and Group 4—high dose STX/TTX daily (325 and 162.5 µg/kg for STX.2HCl and TTX, respectively). At 8.30 am on each day of the 21-day feeding study, mice were weighed to allow the calculation of the exact quantity of STX/TTX required to maintain the desired dose rate. The required volume of STX/TTX solution was then taken and used to prepare the doses as described in Section 4.3. Water was available ad lib. Each day, food consumption was measured, and posture/appearance noted. This observation was carried out with mice in their home cage and included an assessment of movement (level of activity) and appearance (posture, ear position, brightness of the eyes and coat condition). The heart rate and blood pressure of each mouse was measured on days 14 and 21 using a blood pressure analysis system (BP-2000, Visitech Systems, Apex, NC, USA). The motor coordination of each mouse was measured on days 0, 7, 14 and 21 using an accelerating rotarod as previously described. On the same days, the grip strength of each mouse was determined using a grip strength meter (MK3805S, Muromachi, Tokyo, Japan). For both motor coordination and grip strength, mice were tested twice and the results averaged. At the completion of the 21-day test period, all mice were euthanised via CO_2_ inhalation. Using heparin as an anticoagulant, blood samples were collected by heart puncture. Haematocrit values (HCT), haemoglobin levels (HB), mean corpuscular volumes (MCV), mean corpuscular haemoglobin (MCH), mean corpuscular haemoglobin concentrations (MCHC), and red and white blood cell counts were measured in whole blood. In addition, plasma was analysed for activities of aspartate aminotransferase (AST) and alanine aminotransferase (ALT) and for levels of urea, total protein (TP), albumin (ALB), globulin, sodium, potassium, chloride and creatinine (CRN) (IDEXX laboratories, Hamilton). At necropsy, each mouse was examined for any macroscopic changes and the brain, heart, kidneys, liver and spleen collected and weighed. Samples of adrenals, lungs, pancreas, gastrocnemius, jejunum (3 mm section), ovary/uterus or testes, spinal cord (3 × 2 mm sections), stomach (washed), thymus and urinary bladder were also collected, and all were fixed in 4% buffered formaldehyde. These specimens were processed for histological examination and were then examined by the same pathologist, who was blinded to the treatment groups.

### 4.5. Statistical Analysis

The bodyweight, food consumption, motor coordination, grip strength, blood pressure and heart rate data were analysed using linear mixed-effects models (LMMs) fitted by using restricted maximum likelihood (REML). The random model comprised of effects for replicate (i.e., the row in the housing arrangement) and mouse. The basic fixed model comprised of effects for treatment group, day, gender and all two- and three-way interactions. To account for any differences between mice, the data collected on day 0 were used as a covariate. Where appropriate, we corrected for homogeneity violations using constant variance structures.

The haematological, serum biochemical and organ weight data were analysed using LMMs. The model comprised treatment, gender, and their interaction as fixed effects, while row was included as a random effect. To stabilize variance, log transformations were used when necessary.

In all analyses, diagnostic plots (i.e., residual and quantile–quantile plots), and plots of Pearson residuals against the fitted values and versus each explanatory variable in the model were used for model validation. A standard test for temporal autocorrelation on the simulated residuals based on the Durbin–Watson test on the uniformly scaled residuals was used to test the underlying assumption of independence (i.e., temporal correlation) [R package DHARMa, [39]]). For all models, the significance of the fixed terms was assessed using Type II or III (Wald) tests measured by a chi-square statistic for linear mixed-effects models [R package car; Fox and Weisberg [40]], and Fisher’s unprotected least significant differences at the 5% level (LSD (5%)) were used to compare means. Statistical analyses were performed using R version 4.2.0 [41].

## Figures and Tables

**Table 1:** Heart rate and blood pressure of mice dosed with control matrix, 175/87.5 µg/kg STX.2HCl/TTX (low), 250/125 µg/kg STX.2HCl/TTX (medium) or 325/162.5 µg/kg STX.2HCl/TTX (high) daily for 21 days.

Item	Control ^1^	Low STX/TTX ^1^	Medium STX/TTX ^1^	High STX/TTX ^1^
Heart rate (BPM)	614 ± 12.03 ^a^	607 ± 8.82 ^a^	615 ± 11.45 ^a^	616 ± 8.35 ^a^
Systolic BP (mmHg)	107 ± 3.60 ^a^	108 ± 3.64 ^a^	107 ± 3.92 ^a^	110 ± 1.88 ^a^
Diastolic BP (mmHg)	58.1 ± 2.91 ^ab^	58.6 ± 3.42 ^a^	51.1 ± 2.39 ^b^	55.3 ± 2.54 ^ab^

^1^ Values are means ± standard error of the mean. Fisher’s unprotected least significant differences were used to compare the treatment means. Two means that have no letter in common are statistically different at the 5% level.

**Table 2:** Haematology data of mice dosed with control matrix, 175/87.5 µg/kg STX.2HCl/TTX (low), 250/125 µg/kg STX.2HCl/TTX (medium) or 325/162.5 µg/kg STX.2HCl/TTX (high) daily for 21 days.

Item	Control ^1^	Low STX/TTX ^1^	Medium STX/TTX ^1^	High STX/TTX ^1^
HCT (L/L)	0.50 ± 0.01	0.50 ± 0.01	0.49 ± 0.01	0.49 ± 0.02
HB (g/L)	144 ± 1.4	142 ± 1.5	135 ± 1.8	138 ± 5.9
RBC (×10^12^/L)	8.87 ± 0.12	8.91 ± 0.12	8.35 ± 0.08	8.54 ± 0.46
MCV (fL)	56.9 ± 0.34	56.0 ± 0.45	57.3 ± 0.88	57.6 ± 1.53
MCH (pg)	16.3 ± 0.15	16.0 ± 0.00	16.3 ± 0.16	16.4 ± 0.28
MCHC (g/L)	285 ± 2.2	285 ± 2.1	286 ± 3.6	283 ± 2.8
WBC (×10^9^/L)	8.54 ± 0.75	7.87 ± 0.78	7.23 ± 0.90	7.64 ± 0.43
Neutrophil (%)	15.3 ± 0.70	14.2 ± 2.29	12.2 ± 0.60	15.2 ± 1.46
Lymphocyte (%)	75.6 ± 0.85	68.4 ± 8.4	76.5 ± 1.80	76.0 ± 1.90
Monocyte (%)	5.0 ± 0.48	14.6 ± 8.2	8.25 ± 4.19	5.60 ± 0.66
Eosinophil (%)	3.57 ± 0.48	3.86 ±1.43	2.75 ± 0.30	2.80 ± 0.41

^1^ Values are means ± standard error of the mean. Fisher’s unprotected least significant differences were used to compare the treatment means. None were statistically different at the 5% level. HCT, haematocrit value; HB, haemoglobin level; RBC, red blood cells; MCV, mean corpuscular volume; MCH, mean corpuscular haemoglobin; MCHC, mean corpuscular haemoglobin concentration; WBC, white blood cells.

**Table 3:** Serum biochemical data of mice dosed with control matrix, 175/87.5 µg/kg STX.2HCl/TTX (low), 250/125 µg/kg STX.2HCl/TTX (medium) or 325/162.5 µg/kg STX.2HCl/TTX (high) daily for 21 days.

Item	Control ^1^	Low STX ^1^	Medium STX ^1^	High STX ^1^
AST (IU/L)	402 ± 189 ^a^	646 ± 264 ^a^	565 ± 98 ^a^	287 ± 95 ^a^
ALT (IU/L)	123 ± 76 ^a^	304 ± 113 ^ab^	501 ± 197 ^b^	162 ± 74 ^ab^
Urea (mmol/L)	8.20 ± 0.49 ^a^	6.86 ± 0.27 ^a^	7.32 ± 0.20 ^a^	7.48 ± 0.45 ^a^
TP (g/L)	52.4 ± 1.2 ^a^	52.5 ± 0.5 ^a^	50.5 ± 0.9 ^a^	53.6 ± 2.3 ^a^
ALB (g/L)	29.5 ± 0.62 ^a^	30.3 ± 0.58 ^a^	28.5 ± 0.68 ^a^	31.2 ± 1.70 ^a^
Globulin (g/L)	23.3 ± 0.87 ^a^	22.4 ± 0.35 ^a^	22.0 ± 0.55 ^a^	22.8 ± 0.82 ^a^
CRN (µmol/L)	6.8 ± 0.70 ^a^	5.6 ± 0.57 ^a^	8.4 ± 0.94 ^a^	7.3 ± 1.05 ^a^
A/G ratio	1.28 ± 0.05 ^a^	1.35 ± 0.04 ^a^	1.32 ± 0.04 ^a^	1.39 ± 0.06 ^a^
Na (mmol/L)	153 ± 1.0 ^a^	152 ± 1.5 ^a^	153 ± 1.3 ^a^	151 ± 3.1 ^a^
K (mmol/L)	8.31 ± 1.14 ^a^	8.95 ± 1.05 ^a^	8.93 ± 1.13 ^a^	10.17 ± 1.47 ^a^
Cl (mmol/L)	111.8 ± 0.98 ^a^	112.0 ± 1.32 ^a^	113.9 ± 1.89 ^a^	110.0 ± 1.79 ^a^

^1^ Values are means ± standard error of the mean. Fisher’s unprotected least significant differences were used to compare the treatment means. Two means that have no letter in common are statistically different at the 5% level. AST, aspartate aminotransferase; ALT, alanine aminotransferase; TP, total protein; ALB, albumin; CRN, creatinine.

**Table 4:** Organ weights, expressed as percentage of bodyweight, for mice dosed with control matrix, 175/87.5 µg/kg STX.2HCl/TTX (low), 250/125 µg/kg STX.2HCl/TTX (medium) or 325/162.5 µg/kg STX.2HCl/TTX (high) daily for 21 days.

Item	Control ^1^	Low STX ^1^	Medium STX ^1^	High STX ^1^
***Females***				
brain	1.80 ± 0.11 ^ab^	1.83 ± 0.04 ^a^	1.85 ± 0.06 ^a^	1.58 ± 0.18 ^b^
heart ^2^	0.62 ± 0.03	0.58 ± 0.01	0.59 ± 0.02	0.56 ± 0.03
kidneys	1.67 ± 0.17 ^a^	1.44 ± 0.03 ^b^	1.46 ± 0.03 ^b^	1.39 ± 0.03 ^b^
liver ^2^	5.44 ± 0.34	5.12 ± 0.09	5.08 ± 0.19	5.15 ± 0.26
spleen ^2^	0.57 ± 0.02	0.47 ± 0.02	0.49 ± 0.04	0.76 ± 0.27
***Males***				
brain	1.47 ± 0.05 ^a^	1.55 ± 0.03 ^ab^	1.62 ± 0.03 ^ab^	1.69 ± 0.04 ^b^
heart ^2^	0.61 ± 0.02	0.56 ± 0.01	0.60 ± 0.03	0.57 ± 0.02
kidneys	1.83 ± 0.06 ^a^	1.85 ± 0.03 ^a^	1.98 ± 0.04 ^a^	1.92 ± 0.01 ^a^
liver ^2^	5.65 ± 0.07	5.38 ± 0.14	5.70 ± 0.34	5.48 ± 0.06
spleen ^2^	0.39 ± 0.03	0.33 ± 0.01	0.40 ± 0.01	0.36 ± 0.02

^1^ Values are mean ± standard error of the mean. Fisher’s unprotected least significant differences were used to compare the treatment means within each sex. Two means that have no letter in common are statistically different at the 5% level. ^2^ There was no evidence of any interaction of gender and treatment on heart, liver, and spleen weights (*p* > 0.05). The pairwise comparisons between treatments within gender were therefore not conducted.

## Data Availability

The data presented in this study are available on request from the corresponding author.
